# Contributions of Noncanonical Smoothened Signaling During Embryonic Development

**DOI:** 10.3390/jdb5040011

**Published:** 2017-10-17

**Authors:** Tanushree Pandit, Stacey K. Ogden

**Affiliations:** Department of Cell and Molecular Biology, St. Jude Children’s Research Hospital, Memphis, TN 38105, USA; tanushree.pandit@stjude.org

**Keywords:** Sonic Hedgehog, smoothened, signal transduction, development, morphogenesis, noncanonical signaling

## Abstract

The Sonic Hedgehog (Shh) signaling pathway is active during embryonic development in metazoans, and provides instructional cues necessary for proper tissue patterning. The pathway signal transducing component, Smoothened (Smo), is a G protein-coupled receptor (GPCR) that has been demonstrated to signal through at least two effector routes. The first is a G protein–independent canonical route that signals to Gli transcriptional effectors to establish transcriptional programs specifying cell fate during early embryonic development. The second, commonly referred to as the noncanonical Smo signal, induces rapid, transcription-independent responses that are essential for establishing and maintaining distinct cell behaviors during development. Herein, we discuss contributions of this noncanonical route during embryonic development. We also highlight important open questions regarding noncanonical Smo signal route selection during development, and consider implications of noncanonical signal corruption in disease.

## 1. Introduction

During embryogenesis, the Sonic Hedgehog (Shh) signal transduction network instructs cell fate decisions to drive tissue patterning. Corruption of Shh pathway regulation leads to developmental disorders including holoprosencephaly (HPE), Gorlin’s Syndrome, Greig cephalopolysyndactyly, and Pallister-Hall syndrome, and in some cases results in early embryonic lethality [1]. As such, significant effort has been dedicated to understanding how the Shh pathway is activated and how its signal is propagated in ligand-receiving cells.

Vertebrates encode three Hh family ligands: Shh, Desert Hh, and Indian Hh, with Shh being expressed most broadly [2]. All Hh family ligands induce signaling in target cells by binding to Patched (Ptch) membrane receptors. Ptch receptors are twelve-pass transmembrane proteins that share homology with the bacterial Resistance, Nodulation, and Division Transporter superfamily [3,4]. In the absence of Shh, Ptch maintains the signal transducing G protein-coupled receptor (GPCR) Smoothened (Smo) in an inactive state. The mechanism by which Ptch represses Smo signaling is not yet known. However, based upon its relationship to select transporter proteins, the conventional model posits that Ptch controls availability of small molecule modulators of Smo activity [5]. Ptch contains a sterol sensing domain that is required for Smo repression [6], suggesting it may target molecules with sterol rings to control Smo activity. Consistent with this hypothesis, cholesterol and various oxysterols function as direct Smo agonists [7,8,9]. Sterols bind through a ligand binding pocket in the extracellular amino-terminal cysteine-rich domain (CRD) of Smo [10,11,12,13]. A second ligand binding pocket is present in the seven-transmembrane core of Smo, and has been demonstrated to bind natural and synthetic small molecules, endocannabinoids, and the free fatty acid arachidonic acid [14,15,16,17,18].

The precise mechanisms by which activation signals are transduced downstream of Smo have yet to be clearly established. However, it is generally accepted that Smo controls at least two effector routes. The first is the extensively studied canonical signal, which controls the Gli transcriptional effector function. Canonical signaling requires Smo translocation into a sensory organelle called the primary cilium, where it controls proteolytic processing of Gli transcription factors [19,20]. In the absence of a Smo signal, Gli2 and Gli3 are phosphorylated by cAMP-dependent protein kinase (PKA), which tags them for processing into truncated transcriptional repressors [21,22,23]. Ciliary Smo signaling halts Gli processing, allowing for stabilization of Gli2 and Gli3 as full-length transcriptional activators responsible for induction of Shh target genes. One such target is *Gli1*, which functions as a feed-forward activator to sustain or amplify target gene expression [22]. The majority of what is known about how Shh signaling impacts embryonic development stems from study of this effector route. Research focused on ciliary Smo signaling to Gli has been extensively reviewed [2,24,25,26,27] and so will not be discussed here. We will instead focus on Gli-independent Smo signaling, and discuss the comparatively limited body of work centered on contributions of this noncanonical signaling effector route during embryonic development.

## 2. Noncanonical Smoothened Signaling

The specific signaling events downstream of Smo that control noncanonical effectors are not yet clear, but in most cases, are thought to involve GPCR function of Smo [5,26]. Classical GPCRs initiate downstream signaling by activating heterotrimeric G proteins. These protein complexes consist of a guanine nucleotide binding Gα subunit bound to a Gβγ heterodimer. Activation of the heterotrimeric G protein complex occurs in response to ligand-induced conformational shifts of its partner GPCR, which stimulates exchange of GDP for GTP on the Gα subunit [28]. This triggers release of the heterotrimeric G protein complex from the GPCR, and separation of Gα from Gβγ. Gα-GTP and free Gβγ subunits then signal to affect second messengers such as cAMP, cGMP, calcium, inositol triphosphate, diacylglycerol, and select gasses to instruct an appropriate cellular response [29,30,31].

Based upon the stereotypical GPCR topology of Smo, and the prominent role of PKA in regulating Gli repressor formation, it was initially predicted that Smo would signal to Gli through a Gαi heterotrimeric G protein intermediate [32,33]. Active Gαi proteins negatively regulate adenylyl cyclases to reduce intracellular cAMP and inhibit PKA activity, providing a direct route whereby Smo might block Gli repressor formation. Early studies using zebrafish embryos and *Xenopus* melanophores provided indirect support for this functionality [34,35]. A role for Gαi downstream of Smo was first suggested by studies in the zebrafish model system. Injection of mRNA encoding the potent Gαi inhibitor pertussis toxin (PTX) into zebrafish embryos led to patterning defects indicative of compromised Hh signaling. These defects included fusion of eyes, loss of ventral forebrain, and abnormal somite patterning [34]. Effects on somite patterning could be rescued by dominant negative PKA, but not by co-injection of Indian Hh RNA, suggesting PTX action downstream of ligand and upstream of PKA. In *Xenopus*, overexpression of Smo in pigment cells led to persistent pigment aggregation, a phenotype that can result from constitutive Gαi activity [35]. This effect was blocked by expression of dominant negative Gαi or by treatment with Gαi-inactivating PTX, supporting Gαi involvement in Smo-induced pigment aggregation.

Although the aforementioned studies provided circumstantial evidence implicating Gαi action in the Shh signaling cascade, results from in vivo studies aimed at directly testing G protein involvement in Gli regulation failed to reach a consensus. During neural tube development, Shh signaling specifies cell fate by inducing expression of specific transcription factors in discrete domains of the ventral neural tube [2]. In vivo studies aimed at determining whether Gαi modulation in the chick neural tube would alter Shh-induced fate determination suggested that neither inhibition nor constitutive activation of Gαi corrupted neural tube cell fate specification [36]. These results argued against a role for Gαi in regulation of Gli. However, subsequent studies in the chick neural tube, and in *Drosophila,* provided evidence that constitutively active Gαi could trigger phenotypes suggestive of ectopic Gli induction [37,38,39]. Moreover, in vitro interrogation of Smo-G protein coupling revealed that, in the absence of Ptch, Smo constitutively activated all Gαi/o family members including Gαz in a manner that was sensitive to the inverse Smo agonist cyclopamine [40]. Intriguingly, truncation of the intracellular carboxyl-terminal tail of Smo, which is necessary for entry into the primary cilium where it controls Gli processing, did not block Smo-activated Gαi signaling [41]. This finding indicated that signals to G proteins and Gli effectors could be separated, suggesting the existence of two independent Smo signaling arms: a canonical route controlling Gli and a noncanonical route involving Gαi, and controlling Shh-induced cellular responses that occur in a Gli-independent manner. Responses identified as being under the control of noncanonical Smo signaling including cytoskeletal modulation leading to cellular migration and axon guidance, axon fasciculation, neurotransmitter selection, cellular proliferation, and lipid metabolism are discussed in detail below.

## 3. Cytoskeletal Dynamics, Cellular Migration, and Axon Guidance

The Shh-regulated developmental processes most commonly linked to noncanonical Smo signaling are cellular migration and axon guidance, which are related cellular responses that can be regulated through analogous mechanisms [42]. In mouse fibroblasts, Smo signals through Gαi to activate Rac1 and RhoA GTPases, which promote cell migration by modulating cytoskeletal behavior [41]. This signal route is likely active during Shh-induced fibroblast chemotaxis, which has been demonstrated to occur in a Smo-dependent, Gli-independent manner. Although not specifically linked to the Smo-Gαi effector route, Shh-induced chemotaxis was found to be PTX-sensitive, and was maintained in cells expressing a Smo mutant that does not enter the primary cilium or signal to Gli [43]. Smo mutants that fail to enter the primary cilium maintain the ability to couple with Gαi [41], making it feasible that the chemotactic response occurs in a Gαi-dependent manner. Gαi involvement is further supported by the observation that metabolism of arachidonic acid through the 5-lipoxygenase pathway contributes to fibroblast chemotaxis [44]. We recently demonstrated that Smo signaling through Gαiβγ activates the lipid remodeling enzyme cPLA2α to produce arachidonic acid [18], providing a mechanism by which the free fatty acid would be made available for lipoxygenase activity.

Notably, the ability of Smo to affect cell migration through cytoskeletal behavior is not limited to fibroblasts. Smo has been demonstrated to signal though the Gαi-RhoA effector route to promote tubulogenesis of human endothelial cells [45], and to influence the cytoskeleton in osteoblast precursor cells [46]. In osteoblasts, over-activation of the Smo-Gαi signal occurs in response to genetic disruption of primary cilia function, and results in inappropriate induction of stress fibers [46]. These results indicate that ciliary dysfunction can lead to uncontrolled noncanonical Smo signaling, suggesting the balance of canonical and noncanonical signal output is influenced, at least in part, by Smo ciliary localization.

Neuronal cell types represent an additional cellular context in which noncanonical Smo signaling impacts cytoskeletal behavior. In motor neurons, Smo signaling through the 5-lipoxygenase pathway promotes formation of neurite projections, priming the neurons for establishment of proper connectivity at later developmental stages [47]. Shh signaling through Smo has also been shown to promote migration of oligodendrocyte precursors of the optic nerve in both mouse and chick systems [48]. As such, Smo signaling through G protein effectors may be active in multiple neuronal contexts to link Shh signaling with cytoskeletal behavior during development.

**Figure 1:**
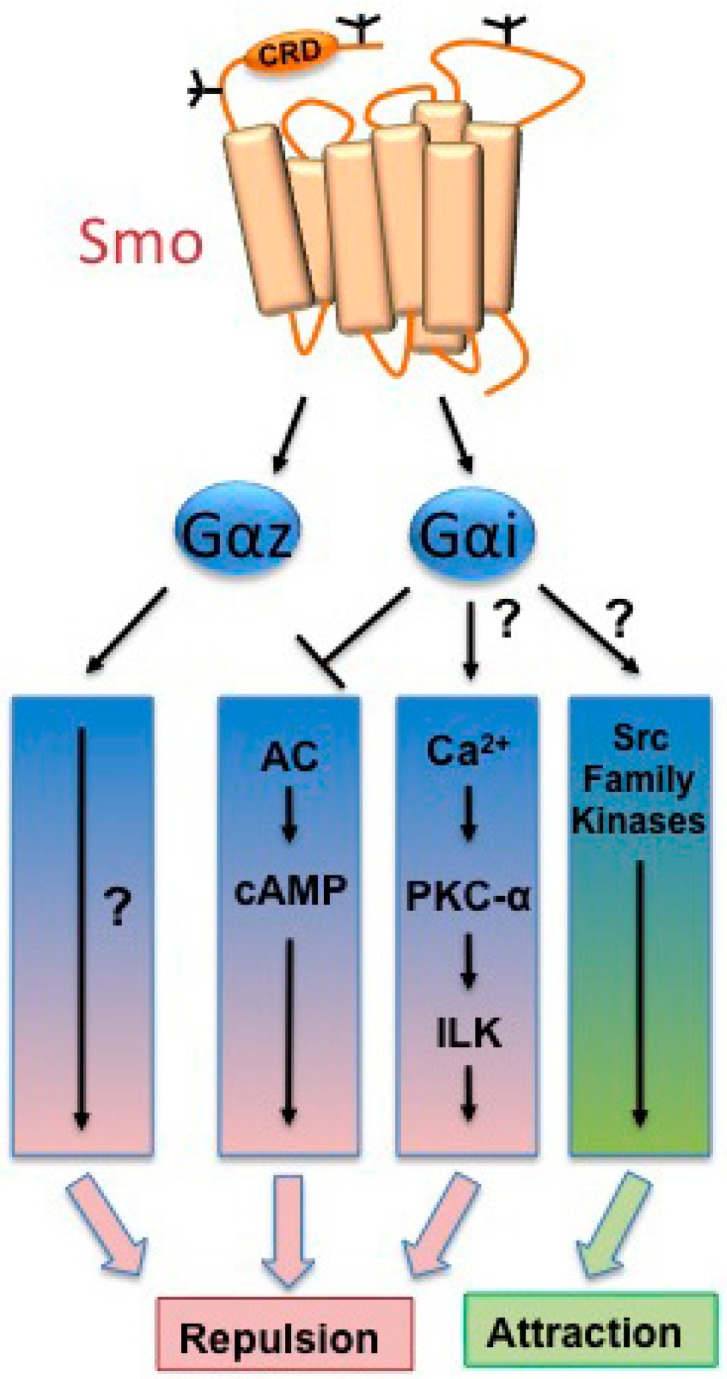
Noncanonical Smoothened signals contributing to axon guidance. The diagram summarizes noncanonical Smo signals that have been reported to influence axon guidance. The mechanism(s) by which Smo signals to modulate intracellular Ca^2+^ levels or control Src Family Kinase activation to influence neuronal axon guidance is not yet established, but we speculate signaling could occur through Gαi. Smo signals through Gαz to repel enteric neurons. Effectors acting downstream of Gαz in this context have not been defined. Repulsion is indicated in pink and attraction is indicated in green. CRD—cysteine rich domain ligand binding pocket. N-linked glycans in the Smo extracellular domain are indicated in black.

**Figure 2:**
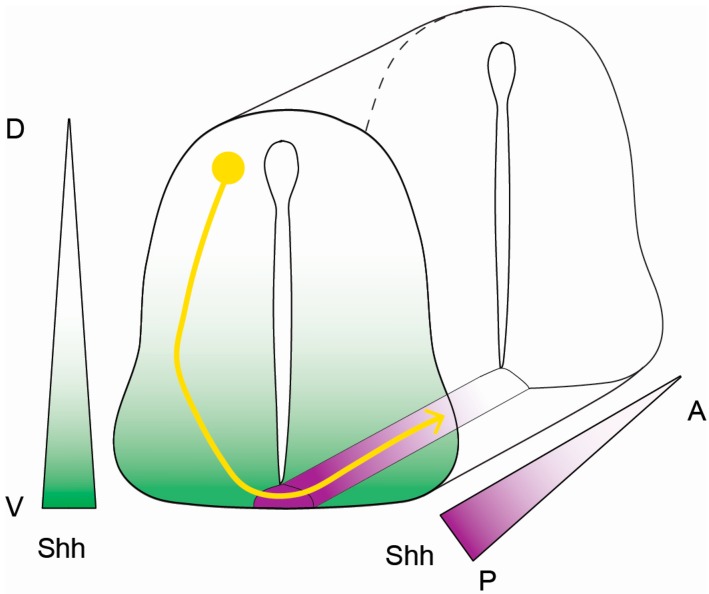
Shh-mediated commissural axon guidance cues. The diagram indicates the trajectory of a commissural axon (yellow) responding to Shh guidance cues in the neural tube. Dorsally (D)-localized dI1 neurons project their axons ventrally (V) toward chemo-attractive Shh (green) that is secreted from the floor plate (magenta). Upon crossing the floor plate, commissural axons change their response to Shh, resulting in axon repulsion. Shh-mediated repulsion prompts axons to target from posterior (P, Shh-high, magenta) to anterior (A, Shh-low, white) to facilitate proper terminal positioning.

Another process involving cytoskeletal rearrangements that has been linked to noncanonical Smo signaling is commissural axon guidance (Figure 1). During spinal cord development, axons encounter attractive and repulsive guidance signals that must be properly integrated to specify an appropriate targeting route. For example, in the neural tube, dI1 dorsal interneurons must first extend ventrally toward the floor plate, cross the midline, and then turn rostrally to establish functional positioning (Figure 2) [49]. A number of guidance cues contribute to this process, including dorsal repulsive factors BMP and Draxin, and ventral attractive factors Netrin, Shh, and VEGF [49]. Shh was first identified as a floor plate-derived attractive cue based upon its ability to reorient axons of spinal cord explants [50]. Genetic and chemical inhibition studies revealed that Shh-mediated attraction was dependent upon Smo. However, the rapid response of axons to Shh suggested a transcription-independent mechanism, rendering Smo signaling through its canonical effector route unlikely. Consistent with this notion, Smo was demonstrated to function in a Gli-independent manner to guide axons [51]. In vitro axon guidance assays revealed that Shh, via Smo, induced phosphorylation and activation of Src family kinases (SFKs) to alter axon trajectories. Although the Shh co-receptor Boc was found to be a necessary upstream component for this functionality, the downstream noncanonical Smo effector route signaling to SFKs to alter the actin cytoskeleton was not identified [51,52]. Smo signaling though Gαi to activate SFKs is feasible, as Gαi family heterotrimeric G proteins have been documented to activate Src though their activated Gβγ subunits [53]. However, SFKs can also be directly activated by select GPCRs without the use of a heterotrimeric G protein intermediate [53], warranting further investigation into the precise mechanisms by which Smo signals to SFKs.

In addition to functioning as an attractant prior to midline crossing, floor plate-derived Shh also functions to repel commissural axons in a Smo-dependent manner post-crossing (Figure 2) [49]. In ovo RNAi experiments in the chick neural tube have suggested the repulsive role of Shh occurs independently of Smo signaling, and instead results from function of Hedgehog interacting protein (Hip) masking the Shh signal [54]. However, more recent work using rat spinal cords demonstrated that Shh signals in a Smo-dependent manner to activate axons for responsiveness to Semaphorins, which mediate repulsion upon midline crossing [55]. The shift to repulsion correlates with a Shh-induced decrease in axonal cAMP concentration, consistent with the established propensity of low cAMP and reduced PKA activity to favor axon repulsion [55,56]. Alterations of axon trajectories that were observed in rat spinal cords following loss of Shh or Smo could be recapitulated by forskolin-mediated cAMP production, which can functionally mimic the loss of Gαi activity [55]. As such, the Smo-Gαi effector route may contribute to commissural axon guidance both pre- and post-midline crossing. However, reduced PKA activity post-crossing has been demonstrated to involve 14-3-3 proteins binding PKA [57]. PKA-14-3-3 binding was found to stabilize PKA regulatory and catalytic subunit association, thereby locking PKA in an inactive state [57]. Function of 14-3-3 in axon repulsion was not determined to be under the control of Shh signaling, suggesting parallel PKA-regulatory pathways likely synergize with Shh to assure proper axon turning post-midline crossing.

Recent work suggests that, in addition to affecting axon guidance in the central nervous system, noncanonical Smo signaling is also utilized in the enteric nervous system to repel enteric axons of the gut. In this context, Smo signaling through the Gαi family member Gαz repels axons to prevent them from projecting into intestinal villi [58]. Gαz is the only Gαi family member that is resistant to PTX and is the family member most potently activated by Smo in in vitro assays [40]. Despite high Smo activity toward Gαz, enteric axon guidance is the only context in which Smo and Gαz have been functionally coupled. Further investigation is needed to ascertain whether Gαz has been overlooked in other contexts due to PTX insensitivity, or because Smo engages Gαz in a limited number of cell types. Intriguingly, Shh-induced enteric axon repulsion could not utilize Boc or Cdo co-receptors, and instead required the Gas1 co-receptor to be present at the enteric axonal terminals to induce the Gαz response [58]. These results may suggest that upstream receptors could contribute to G protein selectivity by Smo in different tissue contexts during development.

## 4. Axon Fasciculation

As axons travel toward their final targets, neighboring axons bundle as they grow, allowing them to track along paths determined by earlier pioneer axons [59]. Although an effect of Shh on fasciculation has not been reported in the neural tube, Shh-mediated cAMP reduction in chick retinal ganglion cell axons has been found to halt growth cone extension to allow for fasciculation of the optic commissure as it crosses the optic chiasm [60]. This response is dependent upon a Shh-induced Ca^2+^ increase that triggers protein kinase Cα and integrin-linked kinase activation [61]. The documented ability of Smo to increase cellular Ca^2+^ concentration through Gαi [62] suggests these responses may occur in response to signaling through its noncanonical Gαi effector route.

## 5. Neurotransmitter Selection

Studies in *Xenopus* support that noncanonical Smo-Gαi signaling regulates post-mitotic neuronal differentiation through controlling neurotransmitter expression [62]. During spinal cord development, determination of the neurotransmitter phenotype is controlled by Ca^2+^ spikes in response to specific extracellular signals [62,63,64]. Shh treatment of *Xenopus* spinal cords has been observed to induce phospholipase C-mediated inositol triphosphate (IP3) production at the primary cilium, leading to the release of Ca^2+^ from internal stores and influx through transient receptor potential cation channel 1 (TRCP1). These calcium transients resulted in the establishment of a GABAergic neuronal phenotype, indicating a role for Shh signaling in post-mitotic neuron differentiation. Importantly, in this study, Shh-regulated Ca^2+^ modulation was found to be inhibited by PTX or constitutively active PKA, indicating that Smo-Gαi signaling is likely required [62].

Subsequent studies in mouse fibroblasts demonstrated the ability of Smo signaling to control ciliary Ca^2+^ by opening TRP Ca^2+^ channels in non-neuronal cell types [65]. The specific channels controlled by Smo are not yet established, but the heterotrimeric TRP channel PKD1-L1/PKD2-L1 has been proposed to modulate Smo-regulated Gli signaling by governing Shh effector ciliary trafficking [66]. In fibroblasts, increased ciliary Ca^2+^ can also inhibit activity of ciliary adenylyl cyclases 5 and 6, leading to decreased cAMP and PKA activity, and increased Gli transcriptional activity. The precise mechanism by which Smo signals to control Ca^2+^ in this cellular context was not determined, but was demonstrated to be PTX-insensitive, suggesting Gαi independence [65]. However, PTX-insensitive Gαz was not tested. As such, future interrogation will be necessary to determine whether Smo-regulated fibroblast ciliary Ca^2+^ modulation occurs through Smo-Gαz coupling or through a novel noncanonical Smo signal.

## 6. Cellular Proliferation

The ability of Shh to induce proliferative responses by activating mitogenic transcriptional programs is well established [67]. Recent studies demonstrate that, in addition to initiating a canonical signal to control cellular proliferation, Smo can signal through Gαi to induce a proliferative response [38,68]. Functional interrogation of Gαi in cerebellar granule neuronal precursors (CGNP) revealed that overexpression of active Gαi enhanced Shh-induced proliferation in this neuronal cell type. Knockdown of Gαi2 or Gαi3 in CGNPs reduced proliferation, supporting a requisite contribution of Gαi to the Shh/Smo induced proliferative response [38]. Similarly, in vivo expression of constitutively active SMOM2 in basal epithelial cells of the mammary gland was observed to induce proliferation of neighboring cells in a Gαi-dependent manner [68]. Chemical ablation of Gli revealed that the proliferative response in mammary epithelial cells was fully independent of Gli function, providing in vivo support for the ability of the noncanonical proliferative signal to occur in the absence of a Shh transcriptional response [68]. The Gαi-activated signal driving proliferation in adjacently localized mammary epithelial cells was not determined. Nevertheless, the ability of Smo-Gαi signaling to drive proliferation in diverse cell types suggests that the signal response may occur in multiple contexts during development to drive tissue morphogenesis.

## 7. Metabolism

An increasing body of work connects canonical and noncanonical Shh signaling with metabolism in both vertebrate and invertebrate systems [69]. Although early reports presented contradictory roles as to whether Shh signaling was a positive or negative regulator of adipogenesis, recent studies confirm that Shh signaling functions to inhibit adipose fate determination [69,70,71,72]. The inhibitory role of the Shh pathway in fat formation was first identified in *Drosophila* [70]. In this study, formation of the fly fat body was found to be inhibited by over-activation of Hh signaling, and promoted by inhibition of the pathway. In vitro studies in adipocytes revealed that these effects were conserved in vertebrate cells, with Shh signaling effectively diverting fate determination of pre-adipocytes towards an osteogenic program [70]. In a subsequent study, a genome-wide screen in *Drosophila* identified the Hh pathway as the top-scoring signaling cascade capable of modulating adipocyte fate determination [72]. This study revealed that Hh signaling modulated triglyceride levels in the fly fat body, and that these effects were conserved in vivo in mice. In both of these studies, effects occurred in a predominantly Gli-dependent manner. Moreover, a recent study revealed a specific Gli-code that is associated with altered lipid metabolism in the liver [73]. However, increasing cAMP levels in pre-adipocytes in vitro can rescue adipocyte differentiation in the presence of excessive Shh signaling [70,72], raising the possibility that a Smo-Gαi-cAMP signal axis may be capable of influencing adipocyte differentiation in coordination with canonical Smo signaling activity. The ability of the noncanonical arm to influence the canonical arm through Gαi activation is feasible; we recently reported that arachidonic acid produced in response to Smo signaling through Gαiβγ directly associates with Smo to promote its ciliary entry and enhance signaling to Gli [18]. As such, activation of the noncanonical arm may function to amplify canonical signaling in select cell types during cell fate specification and tissue morphogenesis.

An aspect of metabolism that is more clearly linked to Shh-Smo-Gαi signaling is the rapid induction of Warburg-like metabolism in adipocytes [74]. This metabolic response is reminiscent of behavior observed in cancer cells that switch from oxidative phosphorylation to aerobic glycolysis in the presence of oxygen. The adipocyte metabolic switch was found to be Smo-dependent, PTX-sensitive, and rapid, ruling out a dependence on Gli transcriptional activity. Rather, the observed metabolic reprogramming occurred as a consequence of Smo signaling via Gαi to open plasma membrane Ca^2+^ channels, leading to phosphorylation-dependent activation of AMPK [74]. Importantly, this response could be induced by traditional Smo agonists, and by inverse agonists of the Gli effector route behaving as partial agonists for Gαi activation. These results revealed that canonical and noncanonical signal output can be functionally uncoupled, suggesting Smo can bias its signal output in response to differential small molecule binding.

## 8. Future Perspectives

The ability of Smo to signal through at least two distinct effector routes provides Shh signaling the opportunity to integrate with additional signaling pathways and impact wide-ranging biological responses during development. Although knowledge of these novel signaling connections is growing, a number of open questions regarding noncanonical Smo signaling have yet to be addressed. Key among them is identification of the molecular mechanisms controlling Smo effector route selection. As discussed above, studies in pre-adipocytes suggest that Smo effector route selection can be modulated through differential small molecule binding [74]. As such, Smo ligand selectivity may vary across cellular or temporal contexts to assure a proper balance of canonical and noncanonical effector activity during embryonic development.

An additional mechanism by which signal bias might be controlled is through post-translational modification of Smo. We recently identified that loss of Smo N-linked glycosylation can shift signaling away from Gαi and toward Gli [75]. We did not determine the mechanism by which de-glycosylation imparted Smo signal bias. However, we speculate that, as is likely the case with differential ligand binding, differential glycosylation shifts the active Smo conformation to favor one set of downstream effectors over another. Given that cellular glycosylation machinery varies across tissues [76], it is possible that the Smo N-glycosylation signature may exhibit tissue specificity. Future studies are needed to determine whether differential Smo N-glycosylation does indeed occur during development, and whether it correlates with overt signal bias.

It is well established in the literature that Smo sub-cellular localization controls its activity. In order to signal to Gli, Smo must traffic to the tip of the primary cilium [19]. Conversely, Smo is competent to signal to Gαi from outside the primary cilium and from the ciliary base [45,46,69]. As such, the speed of Smo ciliary entry and passage through the ciliary transition zone may dictate the duration or amplitude of noncanonical signal output. This hypothesis is supported by the aforementioned studies in osteoblast precursors demonstrating that genetic ablation of primary cilium function shifts Smo toward uncontrolled Gαi activation [46]. A physiological mechanism by which Smo ciliary entry is controlled may be through the modulation of ciliary membrane lipids. Consistent with this notion, the phosphatidylinositol 3-kinase PI3K-C2α has been demonstrated to be essential for Smo ciliary localization [77]. As such, PI3P may be targeted to influence Smo noncanonical/canonical signal transition. Future studies are needed to test this hypothesis, and to identify additional factors influencing Smo ciliary entry and impacting its noncanonical signaling activity.

Continued analysis of noncanonical Smo signaling is essential for a better understanding of the physiological role of Gli-independent Smo signaling during development. Going forward, the impact of noncanonical Smo signaling to disease will also need to be considered. Aberrant Shh signaling is causative in medulloblastoma and basal cell carcinoma, and is commonly activated in a number of additional tumor types [78]. Consequently, the Shh pathway is considered a strong candidate for targeted cancer therapy. Vismodegib, an FDA-approved Smo antagonist, has shown efficacy in the clinic. However, undesirable side effects and emergence of tumor resistance have occurred [79], necessitating improved methods for therapeutic intervention. Given the ability of Smo-Gli inhibitors to behave as partial agonists for Smo-Gαi, Gli-independent Smo effectors will need to be considered for future protocol iterations. As such, continued evaluation of physiological noncanonical Smo signaling in the context of development will be of utmost importance.
